# Effects of Outdoor and Indoor Air Pollution on Respiratory Health of Chinese Children from 50 Kindergartens

**DOI:** 10.2188/jea.JE20120175

**Published:** 2013-07-05

**Authors:** Miao-Miao Liu, Da Wang, Yang Zhao, Yu-Qin Liu, Mei-Meng Huang, Yang Liu, Jing Sun, Wan-Hui Ren, Ya-Dong Zhao, Qin-Cheng He, Guang-Hui Dong

**Affiliations:** 1 Department of Biostatistics and Epidemiology, School of Public Health, China Medical University, Shenyang, Liaoning Province, PR China; 2 Shenyang Environmental Monitoring Center, Shenyang, Liaoning Province, PR China; 3 Institute of Respiratory Diseases, the First Affiliated Hospital of China Medical University, Shenyang, Liaoning Province, PR China

**Keywords:** air pollution, children, respiratory diseases, China

## Abstract

**Background:**

Concentrations of ambient air pollution and pollutants in China have changed considerably during the last decade. However, few studies have evaluated the effects of current ambient air pollution on the health of kindergarten children.

**Methods:**

We studied 6730 Chinese children (age, 3–7 years) from 50 kindergartens in 7 cities of Northeast China in 2009. Parents or guardians completed questionnaires that asked about the children’s histories of respiratory symptoms and risk factors. Three-year concentrations of particles with an aerodynamic diameter ≤10 µm (PM_10_), sulfur dioxide (SO_2_), and nitrogen dioxides (NO_2_) were calculated at monitoring stations in 25 study districts. A 2-stage regression approach was used in data analyses.

**Results:**

The prevalence of respiratory symptoms was higher among children living near a busy road, those living near chimneys or a factory, those having a coal-burning device, those living with smokers, and those living in a home that had been recently renovated. Among girls, PM_10_ was associated with persistent cough (odds ratio [OR]_PM_10__ = 1.44; 95% CI, 1.18–1.77), persistent phlegm (OR_PM_10__ = 1.36; 95% CI, 1.02–1.81), and wheezing (OR_PM_10__ = 1.31; 95% CI, 1.04–1.65). NO_2_ concentration was associated with increased prevalence of allergic rhinitis (OR = 1.96; 95% CI, 1.27–3.02) among girls. In contrast, associations of respiratory symptoms with concentrations of PM_10_, SO_2_, and NO_2_ were not statistically significant among boys.

**Conclusions:**

Air pollution is particularly important in the development of respiratory morbidity among children. Girls may be more susceptible than boys to air pollution.

## INTRODUCTION

The health effects of exposure to ambient air pollutants (eg, particulate matter, sulfur dioxide [SO_2_], and nitrogen oxide [NO_x_]) have been investigated in numerous epidemiologic studies,^1^^–^^7^ which largely agree that exposure to certain ambient pollutants adversely affects public health. However, the available data have limitations, including interference from confounding effects due to ethnic, demographic, and other factors that affect susceptibility to pollution; differences among questionnaires in the definitions of respiratory symptoms and diseases; and lack of appropriate exposure ranges in cross-sectional studies.

Although requirements for environmental protection have significantly improved ambient air quality in China during the past 10 years, air pollution continues to challenge regulatory and health professionals, eg, concentrations of total suspended particulates (TSP) and SO_2_ are still 10 times higher than World Health Organization recommendations. Due to a concomitant increase in motor-vehicle traffic during recent years, traffic-related air pollution poses a serious problem in China. Air pollution in northern cities of China is amplified by the coexistence of soot air pollution and traffic-related air pollution. Therefore, the effects of air pollution on human health need to clarified.

Temporary outdoor levels of particulate matter of 10 µm or less in aerodynamic diameter (PM_10_) and SO_2_ in the province of Liaoning used to be among the highest in China. Current levels still exceed those specified in clean air guidelines, and NO_x_ concentrations have simultaneously substantially increased, albeit with substantial geographical variation. All 3 of these pollutants were consistently low in 1 area near the coast of Dalian and Yingkou cities. This heavy but nonuniform air pollution makes Liaoning well suited for epidemiologic studies; thus, the provincial government of Liaoning funded this study, which was done in 7 cities: Shenyang, Anshan, Benxi, Dalian, Fushun, Liaoyang, and Yingkou. These cities were chosen because they differ widely in inter- and intracity gradients and ambient pollutant levels and therefore offer a valuable epidemiologic opportunity to assess the relationship between exposure and response.

We focused on children aged 3 to 7 years in our study because they spend more time outdoors and exercise more than adults. In addition, children at this age are the most vulnerable, due to their developmental stage of physical growth and lung function. Compared with previous studies performed in China, this study was exceptional because the participants were younger than in previous studies, the number of study sites exceeded those in similar previous studies (facilitating accurate evaluation of the relationship between concentrations of air pollutants and prevalence of respiratory morbidity), and the respiratory health questionnaires used were modeled on those of the American Thoracic Society (ATS).

## METHODS

### Study design

#### Site selection and subject recruitment

This investigation was based on a large-scale epidemiologic study in Liaoning province, which used data from 2009–2010. Liaoning province is located in Northeast China and includes 14 cities. We selected 7 of these cities, to maximize inter- and intracity gradients of the pollutants of interest and minimize the correlation between district-specific ambient air pollutants. On the basis of air pollution measurements recorded at municipal air pollution monitoring stations from 2006 through 2008, investigators selected the cities of Shenyang, Dalian, Anshan, Fushun, Benxi, Liaoyang, and Yingkou form Liaoning province in April 2009. There are a total of 25 districts in the selected 7 cities: 5 in Shenyang, 4 each in the cities of Dalian and Fushun, and 3 each in the cities of Anshan, Benxi, Liaoyang, and Yingkou. In each district, there was only 1 available municipal air monitoring station that collected air pollution data. Two kindergartens within 1000 meters of air monitoring sites were randomly selected from each district (total, 50 kindergartens). Ambient air monitoring stations are required to be located away from major roads, industrial sources, buildings, or residential sources of emissions from combustion of coal, waste, or oil, to ensure reasonable certainty that the results of monitoring reflect background urban air pollution levels rather than pollution from local sources such as traffic or industrial combustion. In all selected districts, both the distance between the station and the kindergartens and the distance between the monitor and participant homes was less than 1.6 kilometers. Within each kindergarten, we identified all children aged 3 to 7 years who had resided in the relevant monitoring district for at least 2 years, yielding a total of 7341 eligible participants from the 50 kindergartens. Before collecting data, a consent form was sent to the parents or guardians to inform them of the survey and request permission for their child’s participation in the survey. All parents or guardians were requested to submit a completed consent form to the local study staff. All procedures in the present study complied with the ethical standards of the responsible committee on Human Experimentation of China Medical University and with the principles outlined in the Helsinki Declaration.

#### Assessment of ambient air pollution exposure

Concentrations of PM_10_, SO_2_, and NO_2_ in 2006–2008 were obtained from municipal air pollution monitoring stations, because these regulated pollutants are routinely measured in all study districts. Measurement of PM_10_, SO_2_, and NO_2_ at the municipal monitoring stations strictly followed the standards established by the State Environmental Protection Administration of China.^6^^,^^8^ Levels of PM_10_ (β-ray absorption method), SO_2_ (ultraviolet fluorescence), and NO_2_ (chemiluminescence) were measured continuously. Daily average concentrations of PM_10_, SO_2_, and NO_2_ were calculated based on data from days for which at least 75% of the valid 1-hour values were available at each station. Yearly mean concentrations were calculated from the arithmetic means of all valid daily mean concentrations during the year. Yearly mean concentration was reported for a station only when at least 75% of daily observations for that year were available. Three-year average concentration was reported for the stations in all 25 districts by averaging the 3 yearly mean concentrations. The mean levels (2006–2008) in each district were computed for each pollutant metric.

#### Questionnaire survey

Cross-sectional information on residential history, lifestyle, household characteristics, and medical histories of children and parents was obtained through a questionnaire survey performed in April 2009. The survey instrument was a standardized questionnaire translated from the ATS Epidemiologic Standardization Project questionnaire,^9^ which was previously shown to be well suited for research such as the present study.^1^^,^^3^^,^^6^^,^^10^ The questionnaire had already been translated into the Chinese, for use in previous studies of other Chinese cities.^1^^,^^3^^,^^6^ Definitions of housing characteristics and environmental tobacco smoke (ETS) exposure in children are shown in Table 1.

**Table 1.** Housing characteristics of participants (*n* = 6730)

Characteristics	Definition	Prevalence%
Coal-burning device	Use of coal-burning device for cooking or heating	3.79
Ventilation device	Use of any of the following ventilation devices: exhaust fan, chimney, or fume hood (typically above a cook stove)	59.29
House pets	Keeping any of the following pets: dog, cat, bird, chicken, duck, or goose	11.60
Home renovation	Any of the following renovations within the previous 2 years: linoleum flooring, painting, new furniture, wall covering, or suspension of ceiling	34.73
Early ETS exposure	Exposure to ETS between birth and age 2 years	49.42
Family history of asthma	Any biological parent or grandparent with diagnosed asthma or bronchial asthma	7.04

ETS, environmental tobacco smoke.

Children were informed of the survey at participating schools. Teachers were given verbal and written instructions, questionnaires with envelopes, and forms to record questionnaire distribution and collection. After obtaining written parental consent for participation, parents were invited to a parent night, with study staff in attendance, where teachers explained the study and the conditions of consent. These questionnaires were filled out by the parents at school or at home. Teachers were instructed not to urge parents to fill out the questionnaire, as compliance was strictly voluntary. Parents who wished to complete the questionnaire at home returned it (via their child) to the teacher in an envelope. All questionnaire responses were recorded electronically in a database according to a standardized code and file structure.

### Definitions of respiratory symptoms and illnesses

Respiratory symptoms were determined based on questionnaire responses, as follows. Persistent cough: answers to several items on coughing indicating that the child had a cough on most days (≥4 days per week) for at least 3 of the previous 12 months, in the absence or presence of cold. Persistent phlegm: answers to several items on phlegm indicating that the child was congested—or brought up phlegm, sputum, or mucus from the chest—on most days (≥4 days per week) for at least 3 of the previous 12 months, in the absence or presence of cold. Asthma symptoms: a positive response to 1 of the following questions, “Has your child ever had an attack of wheezing that caused him/her to be short of breath and had 2 or more such episodes up to the present?”, “Has a doctor ever diagnosed asthma in your child?”, or “When asthma was diagnosed did his/her chest sound wheezy or whistling and was dyspnea also present?”. Current asthma: for a child with a history of asthma, a positive answer to the question “Has your child had an asthma attack during the last 2 years?” or “Does he/she take medicine or receive treatment for asthma or asthmatic bronchitis?”. Wheezing: positive answers to the questions “Does his/her chest ever sound wheezy or whistling, including when he/she had a cold?” and “Has your child had 2 or more such episodes during the preceding 2 years?”. Allergic rhinitis: a positive answer to the question “Has a doctor ever diagnosed allergic rhinitis in your child?”.

### Statistical analysis

The data were analyzed using SAS software (version 9.1; SAS Institute, Cary, NC, USA). We studied the relationship between district-specific ambient levels of pollutants and prevalence rates of the following questionnaire-based morbidity end-points: persistent cough, persistent phlegm, asthma symptoms, current asthma, wheezing, and allergic rhinitis. Stepwise logistic regression was used to determine the association between personal characteristics and each symptom. Any personal covariate that was significant at the level of *P* < 0.15 for a given symptom was included in all subsequent models of pollutant effects for that symptom. To investigate the relationship between symptoms and air pollutants, we used a 2-stage regression approach similar to that used in previous studies.^1^^,^^6^^,^^11^ In the first stage, we fit a single logistic regression model for the condition, including all of the significant personal covariates, and 25 separate intercept terms a*_j_* for each district *j*. These intercept terms represent the logit of the district-specific prevalence rates, adjusted for personal covariates. The adjusted prevalence rate can then be computed as e^a^*^j^*/(1 + e^a^*^j^*). In the second stage model, we regressed the district-specific parameter estimates (a*_j_*, *j* = 1, …, 25) on the district-specific ambient level of a given pollutant, using simple linear “ecologic” regression: a*_j_* = (a*_j_* = α + βZ*_j_* + E*_j_*), where Z*_j_* denotes the pollution variable(s) in district *j*. We expected that if there were a relationship between the condition and pollution, there would be a non-zero slope (β) in this model. The standard *t* test of zero-slope for a regression model was used to determine whether rates of symptoms correlated with pollutants. The quantity e^β^ can be interpreted as the prevalence odds ratio (OR) of symptoms per unit change of a pollutant, adjusted for personal covariates. Both 2006–2008 pollutant levels were tested in these models, and each model considered only 1 pollutant at a time. Analyses were performed on all subjects, and on boys and girls separately. In addition, we translated the values of slopes to prevalence ORs and 95% CIs scaled so that the interquartile range (25th to 75th percentile of district-specific concentrations) corresponded to a 1-unit change.

## RESULTS

### Air pollution levels

Table 2 summarizes the distribution of the various air pollutants studied, based on the 2006–2008 estimate upon which the districts were selected. Generally, for the 2006–2008 estimate, the correlations of NO_2_ with PM_10_ (0.70) and SO_2_ (0.53) tended to be relatively low across the 25 districts (see Supplemental Material, eTable), although the correlation between PM_10_ and SO_2_ (0.78) was higher. District-specific ambient pollutant levels were stable over several years before and during the study.

**Table 2.** Range of 3-year average concentrations of ambient air pollutants in the 25 districts, 2006–2008

Air pollutant	Mean ± SD	Median^b^	Interquartile range^a^	Min	Max
PM_10_ (µg/m^3^)	124.2 ± 24.1	128	31	79	171
SO_2_ (µg/m^3^)	50.3 ± 16.8	50	21	20	80
NO_2_ (µg/m^3^)	36.7 ± 7.6	36	10	21	51

^a^Range from 25th to 75th percentile of district-specific concentrations.

^b^Median range of deviation from 3-year mean within the 25 districts; min and max are ranges in the districts with the smallest and largest range of deviation from the districts’ mean.

PM_10_, particles with an aerodynamic diameter ≤10 µm; SO_2_, sulfur dioxide; NO_2_, nitrogen dioxides.

### Characteristics of participants

Of the 7341 eligible participants, 6730 returned the medical questionnaire (91.68%); 3402 children were male. Participation rates varied across districts (from 81.3% to 94.7%) but did not correlate with pollution levels or disease prevalence. The children included in the analyses were 3 to 7 years of age, and most (78.05%) had been breast-fed. The characteristics of participants, by city, are shown in Table 3.

**Table 3.** Characteristics of participants, by city^a^

	Shenyang	Dalian	Anshan	Fushun	Benxi	Liaoyang	Yingkou
*n*	2244	984	1046	780	606	633	437
Age, mean (SD), years	4.7 (0.9)	4.8 (0.7)	4.8 (1.0)	4.7 (0.8)	4.8 (0.7)	4.7 (0.9)	5.7 (0.6)
Male	51.1	50.5	46.4	51.7	52.0	50.4	54.0
Breast feeding	71.4	82.8	74.6	80.6	89.8	82.9	82.2
Parental education <high school graduate	9.9	8.2	31.2	29.1	11.2	13.3	12.1
Family history of allergy	13.2	19.1	12.2	15.3	17.3	10.3	9.8
Family history of asthma	7.3	10.4	4.4	6.9	9.1	5.1	4.8
House near main road	32.8	46.3	39.7	51.2	49.7	42.5	38.7
House near factory or chimney	12.2	14.1	16.0	21.4	33.3	17.7	13.3
Home coal use	2.0	2.1	3.1	11.8	5.5	3.3	2.8
Ventilation device	68.5	76.5	51.4	37.7	54.5	48.7	52.6
House pets	8.6	11.2	18.0	12.7	9.6	13.6	11.0
Home decoration	42.9	37.6	28.9	19.1	30.7	34.9	33.6
Early ETS exposure	16.4	19.0	24.9	34.4	21.5	21.2	25.2
Current ETS exposure	44.2	49.9	49.1	65.8	51.3	45.3	50.1
Persistent cough	11.9	17.9	7.7	18.1	17.7	11.4	8.7
Persistent phlegm	4.4	7.1	5.2	8.6	5.6	5.1	3.2
Asthma symptoms	8.0	9.6	6.5	8.5	6.4	5.1	6.2
Current asthma	3.8	4.7	1.5	3.9	3.1	3.0	3.4
Wheezing	10.1	12.2	5.1	13.3	14.9	10.7	12.4
Allergy rhinitis	2.9	6.5	2.3	1.5	3.1	1.0	0.9

ETS, environmental tobacco smoke.

^a^Values are percentages unless otherwise specified.

### Health effects

The prevalence rates of persistent cough, persistent phlegm, asthma symptoms, current asthma, wheezing, and allergic rhinitis were 13.1%, 5.5%, 7.5%, 3.4%, 10.6%, and 2.9%, respectively. The prevalence of respiratory diseases was slightly higher in boys than in girls, but the difference was not significant (*P* > 0.05).

Table 4 shows multivariate relationships between risk of respiratory morbidity and potentially relevant personal or residential characteristics. The risk of persistent phlegm was higher among children who lived near a factory or chimney, those whose family had a history of allergy, those whose home had been renovated in the previous 2 years, those whose family had a coal-burning device, and those exposed to passive tobacco smoke at home. Risks were lower for breast-fed children. Patterns for persistent cough were similar but, in addition, children living near a busy road were at higher risk.

**Table 4.** Odds ratios for respiratory morbidity associated with housing and personal characteristics

Characteristics (reference)	Persistent cough	Persistent phlegm	Asthma symptoms	Current asthma	Wheezing	Allergy rhinitis
Sex (male)	1.00	1.09	0.69^b^	0.69^b^	0.82^b^	0.67^b^
Breast feeding (not breastfed)	0.83^b^	0.65^b^	0.70^b^	0.81	0.82^b^	0.89
Parental education (≥high school graduate)	0.95	1.26	1.19	0.94	0.77^b^	1.04
Family allergic history (no history)	2.27^b^	2.01^b^	2.85^b^	3.57^b^	2.74^b^	2.78^b^
Family history of asthma (no history)	1.41^a^	1.39	2.71^b^	3.01^b^	1.91^b^	1.32
House near main road (distance ≥20 m)	1.14^a^	1.05	1.08	1.08	1.09	1.24
House near factory or chimney (distance ≥100 m)	1.26^b^	1.36^b^	1.31^b^	1.08	1.33^b^	0.93
Home coal use (no coal use)	1.44^b^	1.48^a^	0.97	0.73	1.25	1.37
Ventilation device (no use)	0.99	0.97	1.10	1.11	0.89	1.20
House pets (no pet)	0.85	1.08	0.97	0.81	0.89	0.90
Home renovation in past 2 years (no renovation)	1.19^b^	1.30^b^	1.22^b^	1.26^a^	1.27^b^	1.61^b^
Early ETS exposure (no exposure)	1.35^b^	1.54^b^	1.57^b^	1.07	1.36^b^	0.93
Current ETS exposure (no exposure)	1.44^b^	1.45^b^	1.11	0.90	1.19^a^	0.86

ETS, environmental tobacco smoke.

^a^*P* < 0.15, ^b^*P* < 0.05.

Items with superscripts are included in the final adjusted model for this variable and are adjusted for each other; remaining variables are adjusted only for footnoted items, as well as for districts.

Factors like home renovation during the previous 2 years and family history of allergic disease were associated with prevalence of current asthma. There was a similar pattern for asthma symptoms—having a chimney or factory near the home was a significant risk factor. Wheezing was associated with having a chimney or factory near the home, home renovation in the previous 2 years, exposure to passive tobacco smoke, and family history of allergy. Home renovation and family history of allergy increased the risk of allergic rhinitis.

The overall adjusted prevalences of respiratory symptoms reported on questionnaires were analyzed by district. Prevalence rates varied considerably among districts. To further investigate potential air quality/respiratory health relationships, we performed ecologic regression analyses on the district intercept terms (log ORs) from the individual-level models in Table 4. Table 5 shows the results based on the 2006–2008 exposure data. Among girls, PM_10_ was only associated with persistent cough, persistent phlegm, and wheezing. Additionally, NO_2_ concentration was significantly associated with increased prevalence of allergic rhinitis (OR, 1.96; 95% CI, 1.27–3.02) in girls. Among boys, however, respiratory symptoms were not significantly associated with concentrations of PM_10_, SO_2_, or NO_2_.

**Table 5.** Odds ratios (ORs) and 95% CIs for respiratory diseases associated with ambient air pollutants (2006–2008)^a^

Symptom	Pollutant	All subjects		Boys		Girls	
		
		OR^b^	95% CI	OR^b^	95% CI	OR^b^	95% CI
Persistent cough	PM_10_	1.23	1.07–1.42	1.06	0.88–1.29	1.44	1.18–1.77
	SO_2_	0.89	0.75–1.25	0.89	0.73–1.08	0.77	0.63–1.04
	NO_2_	1.10	0.96–1.26	1.18	0.98–1.43	1.02	0.84–1.24
Persistent phlegm	PM_10_	1.20	0.98–1.46	1.05	0.80–1.39	1.36	1.02–1.81
	SO_2_	0.86	0.70–1.05	0.94	0.71–1.24	0.78	0.59–1.04
	NO_2_	1.00	0.82–1.22	0.99	0.74–1.32	1.03	0.78–1.36
Asthma symptoms	PM_10_	1.02	0.87–1.21	0.95	0.76–1.18	1.13	0.87–1.46
	SO_2_	1.10	0.93–1.32	1.15	0.91–1.45	1.05	0.80–1.37
	NO_2_	1.16	0.98–1.38	1.20	0.96–1.51	1.11	0.86–1.45
Current asthma	PM_10_	1.10	0.85–1.42	1.06	0.77–1.45	1.14	0.73–1.78
	SO_2_	1.03	0.79–1.34	0.96	0.68–1.34	1.16	0.75–1.78
	NO_2_	1.21	0.93–1.57	1.14	0.82–1.57	1.33	0.86–2.07
Wheezing	PM_10_	1.35	1.15–1.57	1.37	0.91–1.70	1.31	1.04–1.65
	SO_2_	0.83	0.71–0.96	0.83	0.68–1.03	0.83	0.70–1.04
	NO_2_	0.96	0.82–1.12	0.90	0.73–1.12	1.04	0.83–1.30
Allergic rhinitis	PM_10_	0.97	0.76–1.25	1.14	0.80–1.62	0.82	0.57–1.19
	SO_2_	1.29	0.96–1.73	1.15	0.78–1.70	1.52	0.95–2.44
	NO_2_	1.55	1.17–2.04	1.29	0.89–1.86	1.96	1.27–3.02

^a^Single-pollutant models adjusted for personal and environmental factors. Models for all subjects are adjusted for variables with superscripts in Table 3.

^b^ORs are scaled to the interquartile range for each pollutant (ie, 31 µg/m^3^ for PM_10_, 21 µg/m^3^ for SO_2_, 10 µg/m^3^ for NO_2_). Three models were fit for each symptom or condition, 1 for each pollutant.

PM_10_, particles with an aerodynamic diameter ≤10 µm; SO_2_, sulfur dioxide; NO_2_, nitrogen dioxides.

## DISCUSSION

### Effects of household and personal characteristics

Interestingly, breast-feeding was associated with decreased ORs for persistent cough, persistent phlegm, asthma, and wheezing, and which suggests that breast milk has favorable effects. In contrast, family history of atopic diseases significantly increased the ORs for all 6 symptoms/illness. A more recent study also found that maternal asthma and allergic rhinitis were associated with increased risk of their offspring developing allergic conditions.^12^ Residential proximity to busy roads was associated with respiratory symptoms.^13^^,^^14^ As shown in Table 4, the prevalence of respiratory morbidity increased with increasing proximity of a child’s home to traffic. Therefore, traffic-related air pollution was an important risk factor in respiratory diseases among children. Our results suggest that the presence of a chimney near the home increases the risk of some respiratory symptoms among children. A study by Câra and Sultész showed similar results.^15^^,^^16^ A reason for this finding may be that living near a chimney or a factory increases exposure to air pollutants.

Lower parental education level was associated with lower prevalence rates, perhaps because of survey bias, as households headed by parents with lower education levels have less access to health care facilities and usually underreport symptoms. Children whose parents were non-manual laborers (ie, white-collar workers) appeared more likely to be hospitalized for respiratory diseases than the children of manual laborers (ie, blue-collar workers), possibly because white-collar families had better access to hospital facilities. Generally, white-collar workers have a higher level of education and a much higher income as compared with blue-collar workers. So, to avoid the health effects of air pollution, persons with higher education levels usually move from areas with heavy air pollution to less-polluted areas in a city, which may lead to underestimation of the health effects of air pollution. However, when we analyzed the correlation between air pollution levels and education level, the rate of parents with more than a high-school education did not correlate with air pollution levels (*P* > 0.05 for all comparisons). The correlation coefficients between a parental education greater than a high-school education and PM_10_, SO_2_, and NO_2_ were 0.15, 0.16, and 0.09, respectively.

Presence of a coal-burning device was associated with respiratory symptoms among children, especially persistent cough (*P* < 0.05). A Belarus study showed that respiratory symptoms among children were related to less use of coal-based heating.^17^ A previous study of Chinese children also found that residential coal stoves may impair lung function.^18^ In contrast to our results, previous studies in 4 Chinese^6^ and 6 American cities^19^ found a negative association between coal-burning devices and respiratory symptoms in children. This discrepancy may be due to the younger age of our children. Younger children are more sensitive to environmental exposures, as they are in a critical stage of growth and development of lung function.

Home renovation was significantly associated with some respiratory symptoms in children. A recent study showed that use of renovation materials could lead to high indoor concentrations of formaldehyde, volatile organic compounds, and other chemical pollutants.^20^ Substantial evidence indicates that, among the working population (13–65 years), painters have an increased risk for developing asthma and asthma-related symptoms, as well as other respiratory symptoms.^21^^,^^22^ Paints used in home renovation are likely to emit chemical substances that are similar to those emitted by paints used in professional painting. In the home, exposure levels are highest during and shortly after painting, but low levels of exposure may persist for several months. Wood furniture (both painted and varnished) and new furniture are likely to emit chemical substances. Also, furniture, painting, and wall coverings used as exposure indicators are a heterogeneous group of potential emitting materials. The current findings suggest that further attention should be focused on the type of materials used in home renovation.

Some cross-sectional studies reported negative associations between passive smoking and respiratory symptoms in children^23^^–^^25^; the authors suggested that tobacco exposure might also provide protective effects for childhood atopic diseases via selection mechanisms. However, in our study, children exposed to passive smoke had significantly higher risks for persistent cough, persistent phlegm, and wheezing, as in previous studies.^26^

### Effects of ambient air pollution

Air pollution may significantly impair lung function.^5^^,^^27^^,^^28^ As in earlier studies, outdoor exposure to air pollution was associated with prevalence of respiratory diseases when we defined exposure over 3 fairly recent years. Hoek et al reported that long-term exposure to air pollution, as defined by PM_10_ concentration, was associated with increased respiratory symptoms.^29^ A study of 4 Chinese cities also showed that exposure to ambient particulate matter (PM_2.5_ and PM_10_) was associated with decreased development of lung function.^30^ A recent population-based nested case-control study found that prevalence of respiratory symptoms was associated with increased NO_2_ concentration.^4^ The results of a Taiwanese study showed that a 8.6-ppb increase in NO_2_ was associated with an OR of 1.42 (95% CI, 1.21–1.66) for asthma in children.^24^ Another study from Taiwan showed that prevalence of respiratory symptoms with asthma was related to NO_2_ level (OR = 1.81 per 8.79-ppb change; 95% CI, 1.14–2.86).^1^ In contrast to our results, the results of another Chinese study showed no relationship between ambient NO_2_ level and prevalence of respiratory morbidity in children.^3^^,^^6^ A study of 12 communities in southern California also found no association between ambient air pollutants (PM_10_ and NO_2_) and respiratory symptoms in children.^10^

Many previous studies found that girls were more susceptible than boys to environmental exposures.^31^^,^^32^ A cohort study of schoolchildren in Japan found a significant association between prevalence of respiratory symptoms and NO_2_ among girls but not among boys.^33^ A study of 6 cities reported a stronger association between home indoor NO_2_ and respiratory symptoms among girls (OR, 1.7, 95% CI, 1.3–2.2) than among boys (OR, 1.2, 95% CI, 0.9–1.5).^34^ A recent study found that girls are more susceptible than boys to air pollution.^11^ However, Pershagen et al^35^ found no difference between boys and girls regarding the effects of environmental exposure, and, in a Swedish study, the risk of respiratory symptoms related to environmental factors was very similar among males and females.^36^ In our study, associations of PM_10_, SO_2_, and NO_2_ concentrations with the prevalences of respiratory symptoms were statistically significant among girls but not among boys. The mechanism of this female susceptibility is not known and warrants further study. A possible explanation is that girls are physically weaker than boys and consequently more susceptible to the effects of outdoor pollutants.

Our study has some limitations. We evaluated prevalence rather than incidence of respiratory symptoms among children aged 3 to 7 years. Some of the factors studied might have affected the prevalence of respiratory symptoms via effects on disease duration rather than disease incidence. Changes in environmental exposures may have resulted in bias. However, our findings are of considerable interest regardless of whether the observed associations were caused by the effects of incidence or duration.

In conclusion, this large epidemiologic study of Chinese children confirmed that indoor air pollution from home renovation, coal stoves, passive smoking, and other risk factors was harmful to the respiratory system. Outdoor PM_10_, SO_2_, and NO_2_ concentrations were associated with the prevalences of respiratory symptoms among girls but not among boys, which suggests that girls may be more susceptible than boys to outdoor air pollution.

## ONLINE ONLY MATERIALS

eTable. Distribution of 3-year average concentrations (µg/m^3^) of air pollutants in 25 districts of 7 cities in Liaoning province, 2006–2008.
