# Supplementary material for: Effects of Outdoor and Indoor Air Pollution on Respiratory Health of Chinese Children from 50 Kindergartens
Source: J Epidemiol. 2013 Jul 5;23(4):280–7. doi: 10.2188/jea.JE20120175 (PMC3709542; doi:10.2188/jea.JE20120175)
Supplement: eTable. [file je-23-280-s001.pdf]

eTable. Distribution of 3-year average concentrations (μg/m^3) of air pollutants in 25 districts of 7 cities in Liaoning province, 2006-2008.

City	District	PM_{10}	SO_2	NO_2
Shenyang	1	133	51	32
	2	123	44	36
	3	116	42	42
	4	145	78	45
	5	134	58	38
Dalian	6	142	61	44
	7	159	68	51
	8	134	52	46
	9	91	50	33
Anshan	10	126	78	31
	11	137	64	40
	12	120	48	32
Benxi	13	142	46	45
	14	127	41	47
	15	155	62	41
Fushun	16	171	80	42
	17	133	69	37
	18	128	37	33
	19	87	37	30
Liaoyang	20	89	30	36
	21	91	27	33
	22	106	42	24
Yingkou	23	100	20	26
	24	137	50	33
	25	79	23	21

PM_{10}, particles with an aerodynamic diameter ≤10 μm; SO_2, sulfur dioxide; NO_2, nitrogen dioxides.
